# Processing citizen science- and machine-annotated time-lapse imagery for biologically meaningful metrics

**DOI:** 10.1038/s41597-020-0442-6

**Published:** 2020-03-26

**Authors:** Fiona M. Jones, Carlos Arteta, Andrew Zisserman, Victor Lempitsky, Chris J. Lintott, Tom Hart

**Affiliations:** 1 Department of Zoology, University of Oxford, 11a Mansfield Road, Oxford, OX1 3SZ UK; 2 Department of Engineering Science, University of Oxford, Parks Road, Oxford, OX1 3PJ UK; 3 Samsung AI Center, Butyrskiy Val Ulitsa, 10, Moscow, Russia, 125047 & Skolkovo Institute of Science and Technology (Skoltech), Bolshoy Boulevard 30, bld. 1, Moscow, 121205 Russia; 4 Zooniverse, Department of Physics, University of Oxford, Denys Wilkinson Building, Keble Road, Oxford, OX1 3RH UK

**Keywords:** Population dynamics, Software, Conservation biology, Behavioural ecology, Time-lapse imaging

## Abstract

Time-lapse cameras facilitate remote and high-resolution monitoring of wild animal and plant communities, but the image data produced require further processing to be useful. Here we publish pipelines to process raw time-lapse imagery, resulting in count data (number of penguins per image) and ‘nearest neighbour distance’ measurements. The latter provide useful summaries of colony spatial structure (which can indicate phenological stage) and can be used to detect movement – metrics which could be valuable for a number of different monitoring scenarios, including image capture during aerial surveys. We present two alternative pathways for producing counts: (1) via the Zooniverse citizen science project *Penguin Watch* and (2) via a computer vision algorithm (*Pengbot*), and share a comparison of citizen science-, machine learning-, and expert- derived counts. We provide example files for 14 *Penguin Watch* cameras, generated from 63,070 raw images annotated by 50,445 volunteers. We encourage the use of this large open-source dataset, and the associated processing methodologies, for both ecological studies and continued machine learning and computer vision development.

## Abstract (summary)

**Table:** 

Measurement(s)	number of penguins per image • nearest neighbour distance • movement of individuals in images
Technology Type(s)	machine learning
Factor Type(s)	geographic location • time of data collection
Sample Characteristic - Organism	Pygoscelis papua • Pygoscelis antarcticus • Pygoscelis adeliae
Sample Characteristic - Location	Antarctic Peninsula • South Shetland Islands • South Georgia and South Sandwich Islands

Machine-accessible metadata file describing the reported data: 10.6084/m9.figshare.11807325

## Background & Summary

Seabird population changes are considered a reflection of changes within the marine environment, making seabird species key indicators of ecosystem health^1,2^. The penguins (Family: Spheniscidae) are amongst the most threatened, facing environmental stressors such as invasive species, overfishing, pollution and climate change^1–3^. In the Southern Ocean, two key threats – sea ice change^4,5^ and an expanding krill fishery^6^ – have the potential to act synergistically, with a longer ice-free season facilitating a longer harvest^7^. In order to implement effective mitigation strategies, rigorous monitoring of penguin colonies is required. However, the challenges of on-ground monitoring in Antarctica (logistical, practical and financial) have left gaps in data surveys, and the majority of monitoring has occurred on a small scale^8^.

Time-lapse cameras offer a solution to these challenges, and Newbery and Southwell^9^ pioneered the use of automated cameras for penguin monitoring. A large, collaborative network of remote time-lapse cameras now exists in Antarctica, and in Jones *et al*. (2018) we discuss the *Penguin Watch* project, which currently operates over 90 cameras in the region^10^. These cameras are located in the Falkland Islands, South Georgia, the Antarctic Continent and the South Sandwich Islands, and at a minimum usually capture images once per hour year-round between the hours of 0700 and 2000^10^. The camera units (Reconyx HC500 Hyperfire Trail Cameras) are serviced once per year if conditions allow, and are powered either by 12xAA lithium ion batteries or an external rechargeable battery connected to a solar cell^10^.

Owing to the wealth of image data collected, expert manual annotation is unfeasible. Instead, two parallel methods have been employed to efficiently identify penguins in photographs, using – (1) citizen science^10^, and (2) computer vision^11^. The *Penguin Watch* citizen science project (see Jones *et al*. (2018); www.penguinwatch.org), hosted by the *Zooniverse* online platform (www.zooniverse.org), was launched in September 2014. The project tasks volunteers with identifying individual penguins in randomly presented time-lapse images, and categorising them as either an ‘adult’, ‘chick’ or ‘egg’^10^. There is also the option to assign an individual or object to ‘other’, which allows classification of other fauna, humans, or ships^10^. To date, over 6.5 million classifications have been made by roughly 50,000 registered volunteers and numerous anonymous (unregistered) participants.

In Jones *et al*.^10^ we describe a dataset (hereafter ‘Dataset 1’) of 63,070 raw *Penguin Watch* time-lapse images from 14 different cameras, which is stored in the Dryad Digital Repository^12^ (please also see Jones *et al*.^13^ – an erratum correcting for a duplicate dataset). Also in the repository are metadata (such as date/time and temperature information), raw anonymised citizen science classifications (i.e. xy coordinate data for each ‘click’ on a photograph), and ‘consensus click’ data, for each image^12^. The latter are produced via a clustering algorithm^10,14^ – multiple volunteers (a total of ten if the photograph contains animals) annotate each image, meaning a ‘consensus click’ (average xy coordinate) must be generated for each penguin.

This publically available data collection^12^ serves as a resource for ecological researchers, comprising images and citizen science annotations for multiple penguin colonies between March 2012 and January 2014^10^. In addition, *Penguin Watch* volunteer clicks have been employed to train the machine-learning algorithm *Pengbot –* the second annotation method – which detects penguins in raw images using pixel densities^11^.

Whichever approach is adopted, the data must be further processed in order to extract biologically meaningful metrics such as population counts and measures of movement. Here we present a second open-source database in the Dryad Digital Repository (hereafter ‘Dataset 2’^15^), which contains the following files for each of the 14 cameras described in Jones *et al*. (2018): (1) using citizen science: *Kraken Files* (filtered ‘consensus click’ data combined with metadata) and *Narwhal Files* (count data, ‘nearest neighbour distance’ measurements, and metadata); (2) using computer vision: *Pengbot Count Files* (count data). Also included in the repository are the *Penguin Watch Manifest* (image metadata), summary graphs, animations, *Pengbot Out Files* and *Pengbot Density Maps*. The pipelines used to produce these files are presented (in the form of scripts written in the *R* programming language (currently v3.6.0^16^); see Code Availability). These scripts have the potential to be applied to a broad range of data types. For example, a metric of movement, calculated using ‘nearest neighbour distances’, could be useful for analysis of images captured during aerial surveys in subsequent studies.

## Methods

**Fig. 1:**
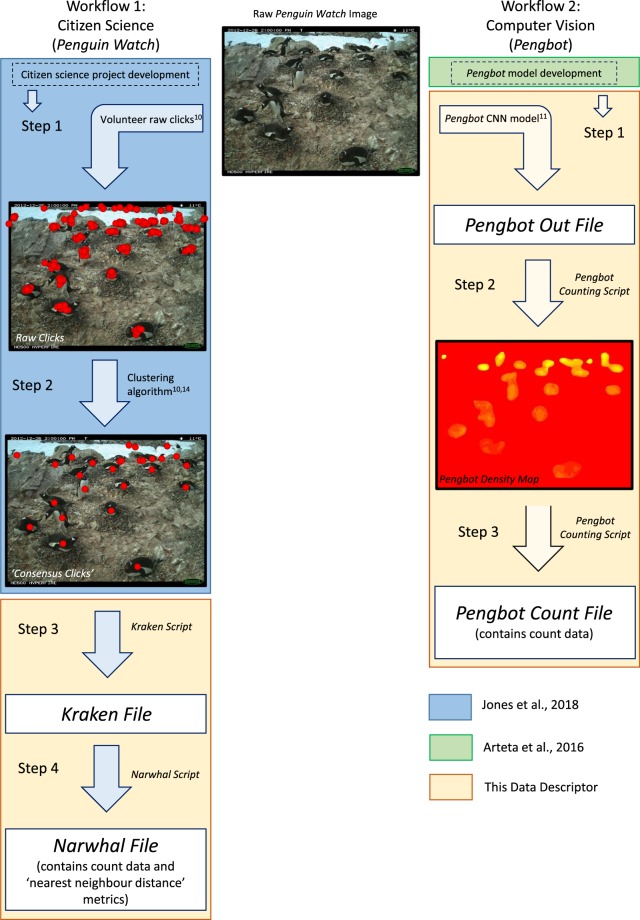
Flow diagram to show the two data processing pipelines, using citizen science (the *Penguin Watch* online project) and computer vision (*Pengbot*). ‘Workflow 1’ (left): images are annotated using citizen science^10^ and aggregated using hierarchical agglomerative clustering^14^. The resulting ‘consensus clicks’ are used to produce the *Kraken Files* and *Narwhal Files* presented alongside this Data Descriptor. ‘Workflow 2’ (right): the *Pengbot* algorithm^11^ is used to produce the *Pengbot Out Files*, *Pengbot Density Maps* and *Pengbot Count Files* presented alongside this Data Descriptor.

In Jones *et al*. (2018) we outline the methodology associated with the *Penguin Watch* remote time-lapse camera network^10^. Here we present the ‘next steps’ in the data processing pipeline, and offer two alternative workflows for extracting count data (i.e. the number of penguins per image): (1) via citizen science (Fig. 1 – left) and (2) via computer vision (Fig. 1 – right). Future studies will examine these count data across colonies and seasons. By comparing count data from the same camera across a number of years, and between colonies, patterns of population change may be identified, which can provide evidence to inform conservation policy decisions. These count data may also be used to determine phenological parameters such as adult arrival and departure date. Changes in phenological timings across years can indicate environmental change^17^, with implications for future population health.

The citizen science workflow is also used to calculate ‘nearest neighbour distances’, a metric which can be used to examine colony spatial structure and movement. This in turn could allow automated detection of behaviour and phenological stage – for example, reduced average chick ‘second nearest neighbour distance’ (see below) could indicate huddling, a behaviour which occurs during the post-guard (crèche) phase^18^. Therefore, these spatial metrics are also useful for population monitoring purposes.

Access to two alternative processing methods (i.e. citizen science and computer vision) is advantageous for a number of reasons. Firstly, certain images – for example photographs partially obscured by snow – may be more suitable for analysis using citizen science, while others – such as those containing high numbers of (uncrowded) individuals – may be more appropriate for computer vision annotation. Furthermore, citizen science is a useful public engagement and educational tool^19^, while computer vision is useful for batch processing large quantities of data which may be considered ‘less interesting’ for human participants (e.g. winter images which contain few animals). Finally, citizen science classifications can be used to train future machine learning algorithms, and the counts produced by each can be cross-validated, ensuring the analyses remain reliable. In fact, Wright *et al*. (2017) demonstrate that a combination of methods (i.e. citizen science and machine learning) can outperform either method used alone^20^.

### The citizen science pipeline – *Penguin Watch*

#### Steps 1 & 2

Raw images are annotated by volunteers; annotations are clustered (Jones *et al*., 2018; see Fig. 1, left, ‘Step 1’ and ‘Step 2’).

The methodology behind the *Penguin Watch* citizen science project is discussed in Jones *et al*. (2018). One of the fundamental paradigms of all such projects is that multiple volunteers process each subject (e.g. image or audio clip), meaning average annotations can be extracted, and errors minimised, through filtering. In Jones *et al*. (2018) we therefore include discussion of a clustering algorithm^14^ which uses agglomerative hierarchical clustering to take the xy coordinates of multiple volunteer clicks and produce a single ‘consensus click’, representing a single individual (penguin or ‘other’).

#### Step 3

Clustered annotations are filtered, and stored in *Kraken Files*.

Here we present the next stage in the data processing pipeline (Fig. 1, left, ‘Step 3’), taking these ‘consensus clicks’ and filtering them, to improve data reliability^10^. The *Kraken Script* (see Code Availability) is used to achieve this, and the results (filtered ‘consensus clicks’ and associated metadata) are stored in *Kraken Files* (Dataset 2^15^). The *Kraken Script* extracts the ‘consensus clicks’ (Dataset 1^12^) and metadata (*Penguin Watch Manifest*; Dataset 2^15^) associated with the camera of interest, and merges them into a single file. In order to be extracted however, an adult ‘consensus click’ must have been generated from four or more raw volunteer clicks. The threshold is lower for chicks and eggs: two or more volunteer clicks must have formed the ‘consensus click’. These rules can be modified within the *Kraken Script* to allow for different filtering thresholds, but we employ these levels as they were found to produce counts most similar to the gold standard^10^. The lower threshold level of *‘num_markings* >* 1’* has also been used for eggs, because we believe they are often missed by volunteers, but a baseline level of filtering must be implemented to eradicate erroneous clicks.

#### Step 4

Penguin counts and ‘nearest neighbour distances’ are calculated (see Fig. 1, left, ‘Step 4’).

**Fig. 2:**
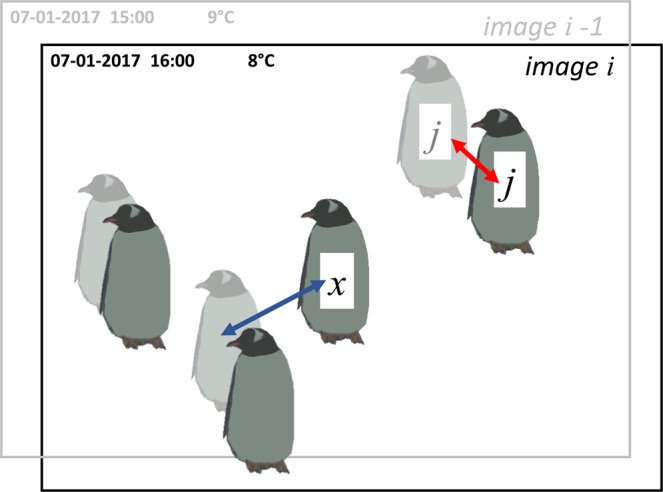
Diagram to illustrate how movement between images is calculated using ‘nearest neighbour distances’. This figure shows two images, image *i* (black text and bold penguins) and image *i-1* (i.e. the previous image; grey text and faded penguins). If the coordinates of adult *j* (image *i*) are appended to the coordinates of the penguins in image *i-1*, adult *j* has a new nearest neighbour (in this case, adult *j* – itself – in its previous position). This ‘nearest neighbour distance’ (shown by the red arrow) therefore represents the distance adult *j* has moved between the two images. However, if an individual moves significantly between photographs (see adult x, which appears only in the later image) the ‘nearest neighbour distance’ (blue arrow) will not represent the individual’s movement. Therefore, this metric is best used when movement is limited, for example during the incubation and guard phase.
- Movement of chicks (as above, for chicks).

*Kraken Files* are the input file type for the *Narwhal Script* (see Code Availability), which produces *Narwhal Files*. These files (Dataset 2^15^) include penguin counts, listed separately for ‘adult’, ‘chick’ and ‘egg’, and ‘nearest neighbour distance’ metrics. Looking at how average ‘nearest neighbour distances’ change over the breeding season is a useful indicator of movement, which in turn can signal the start of a new phenological stage, such as chick crèche. We also attempt to calculate the movement of each individual between images, again using ‘nearest neighbour distances’. The metrics calculated are:

- Number of adults, chicks and eggs.
- Average adult ‘nearest neighbour distance’ (the mean distance between each adult in image *i* and its nearest adult neighbour).
- Average chick ‘nearest neighbour distance’ (the mean distance between each chick in image *i* and its nearest chick neighbour).
- Average chick ‘second nearest neighbour distance’ (the mean distance between each chick in image *i* and its second nearest chick neighbour). Since *Pygoscelis* species lay two eggs, a chick’s first nearest neighbour is likely to be its sibling in the nest. Therefore, looking at second nearest neighbour is a more useful indication of whole-colony movement.
- The standard deviations of each ‘nearest neighbour distance’ metric listed above.
- Movement of adults (see Fig. 2; the mean distance between each adult [*j*] in image [*i*] and its nearest neighbour in image [*i-1*]. This means the coordinates of adult [*j*] are appended to all the adult coordinates in the previous image, to make a new coordinate set ([*i-1*] with one additional data point – adult [*j*] in image [*i*]). The same adult will thus be represented by two data points in this new set of coordinates. ‘Nearest neighbour distance’ is then calculated for the adult of interest [*j*]. It is likely that adult [*j*]’s nearest neighbour will be itself, in its previous position, meaning this value is equivalent to the distance adult [*j*] has moved between images. The mean is calculated to give the average distance moved by all adults in the image.)

This approach to quantifying movement is only robust when photographs are captured within a relatively short time period (*Penguin Watch* photos are generally taken every hour within a set daytime period) and movement is limited; i.e. if an individual moves too far (or another individual comes very close), then its nearest neighbour in image *i-1* will not be itself, but a different individual (Fig. 2). Therefore, this metric is most reliable in the period between egg lay and the end of the guard phase, when individuals are generally found at the nest. However, since individual penguins cannot be tracked using coordinate clicks – or indeed even in time-lapse photographs unless there are distinctive markings (e.g. in African penguins) – this remains a useful and novel indicator of movement. Furthermore, detection of ‘change-points’ is often more important than movement per se. By taking the average of all the movement in an image, these can be identified, and can provide a starting point for determining phenological dates.

An explanation of the different variables and commands found within the *Narwhal script* is provided in Online-only Table 1.

#### Step 5

*Narwhal Files* are used to create plots and animations.

The *Narwhal Plotting Script* (see Code Availability) takes the *Narwhal Files* and creates plots of cumulative summary statistics for each time-lapse image: abundance (for adults and chicks), chick ‘second nearest neighbour distance’, and mean adult ‘nearest neighbour distance’ between the *i*th and (*i-1*)th image (all moving averages). We have grouped these graphs with their associated raw time-lapse image to complete each plot, and created a *Penguin Watch Narwhal Animation* for each camera (Dataset 2^15^) using the open source software GIMP (v2.8)^21^ and VirtualDub (v1.10.4)^22^, to allow easy visualisation of population trends.

### The computer vision pipeline – *Pengbot*

#### Step 0

The *Pengbot* computer vision algorithm – development (Arteta *et al*., 2016).

Within *Penguin Watch* time-lapse images, penguins are often occluded and there can be a high degree of scale variation. This presents a challenge to computer vision developers, and makes simple counting-density estimation algorithms and binary object detectors insufficient for the task of producing reliable counts^11^. Furthermore, variation in camera angle and distance from a colony, obscuration owing to snow or ice, and shape similarity between the objects of interest (i.e. penguins) and background objects (e.g. rocks), all combine to make automatic detection a complex problem^11^.

The dot annotations (raw xy coordinate ‘clicks’; Dataset 1^12^) of *Penguin Watch* volunteers offer a large training dataset for machine-learning algorithms^10^, yet bring their own complications. For example, there is substantial variation amongst the annotations of different volunteers, meaning any model must be robust to noisy labels^11^. Arteta *et al*. (2016) present the first solution to the problem of counting from crowd-sourced dot annotations – the *Pengbot* model. The learning architecture of the *Pengbot* model is a deep multi-task convolutional neural network (CNN)^11^. It makes use of the variability in raw markings by using it to (1) estimate the ‘difficulty’ of an image, and (2) make inferences about local object scale (an individual penguin can measure fewer than 15 pixels or in excess of 700 pixels)^11^.

When the neural network is learning from the images, it tries to learn, among other things, which visual patterns belong to penguins. The optimal way to train it would therefore be to provide complete information regarding every pixel – i.e. which image pixels are part of an object (in this case a penguin) and which are not. This is extremely expensive – both in terms of experts’ time and (if professional annotators are paid) financially – therefore the algorithm relies instead on the assumption that volunteers are very likely to click on penguins. Thus, the more clicks that are used, the more complete the information regarding what ‘penguin pixels’ look like. For this reason, the algorithm is trained on volunteer raw clicks, as opposed to ‘consensus clicks’. There is a step of the learning process that uses a form of count consensus derived from the raw clicks. However, such consensus is defined over the continuously changing definition of what a penguin is (i.e. as the learning progresses), meaning raw clicks must be available all the time.

#### Step 1

The *Pengbot* model is used to generate *Pengbot Out Files*.

The *Pengbot* CNN and associated code are publically available (see Code Availability). Running the algorithm on raw *Penguin Watch* images produces *Pengbot Out Files* (Dataset 2^15^). These are MATLAB files which contain matrices comprising ‘penguin densities’ for each pixel in the photograph. These matrices are visually represented in *Pengbot Density Maps*.

#### Step 2

*Pengbot Out Files* are used to produce *Pengbot Density Maps*.

**Fig. 3:**
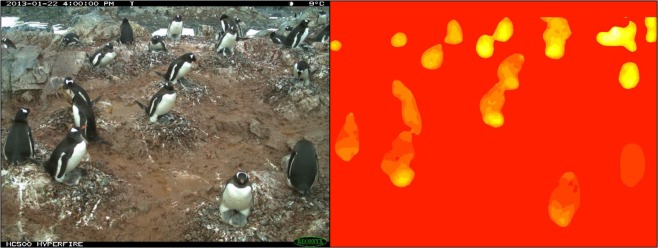
Image data from a Gentoo Penguin (*Pygoscelis papua*) colony at George’s Point, Antarctic Peninsula. Left - *Penguin Watch* time-lapse image GEORa2013b_000153, captured by camera GEORa on 22/01/2013 at 16:00:00; right - the corresponding *Pengbot Density Map*, generated by the *Pengbot* convolutional neural network (CNN).

The *Pengbot Counting Script* (see Code Availability) is used to produce a *Pengbot Density Map* (see Fig. 3, right) from each *Pengbot Out File*, for each raw photograph. The lighter (yellow) parts of the image represent pixels that the algorithm interprets as ‘penguin’, as opposed to background (e.g. snow, ice and rocks). The brightness of these pixels corresponds to a scale of density, such that summing the pixels belonging to a single penguin should give approximately one. In general, pixels towards the top of the image (i.e. in the distance) are brighter – of a higher ‘penguin density’ – than those in the foreground. This is because penguins appear smaller towards the top/back of the image, so fewer pixels are required to comprise a whole individual. Conversely, duller pixels in the foreground have a lower ‘penguin density’, and more are required to count one individual. This method allows the oblique angle and vantage point of the camera to be taken into account when counts are generated from the density maps.

#### Step 3

*Pengbot Count Files* are generated using the density data.

The *Pengbot Counting Script* (see Code Availability) is also used to calculate the number of penguins in each photograph, by summing pixel densities. Note that these counts are for the total number of all individuals, as the model cannot currently distinguish between adults and chicks. The data are stored in *Pengbot Count Files* (Dataset 2^15^).

## Data Records

In Jones *et al*. (2018) we describe a dataset uploaded to the Dryad Digital Repository (Dataset 1^12^) comprising 63,070 unique raw time-lapse images captured by the *Penguin Watch* remote camera network, metadata for these photographs (e.g. date/time and temperature information), raw anonymised classifications from the *Penguin Watch* citizen science project, and ‘consensus clicks’ derived from these classifications^10,13^. Please refer to Jones *et al*. (2018 & 2019) for full details and an explanation of these data types.

**Fig. 4:**
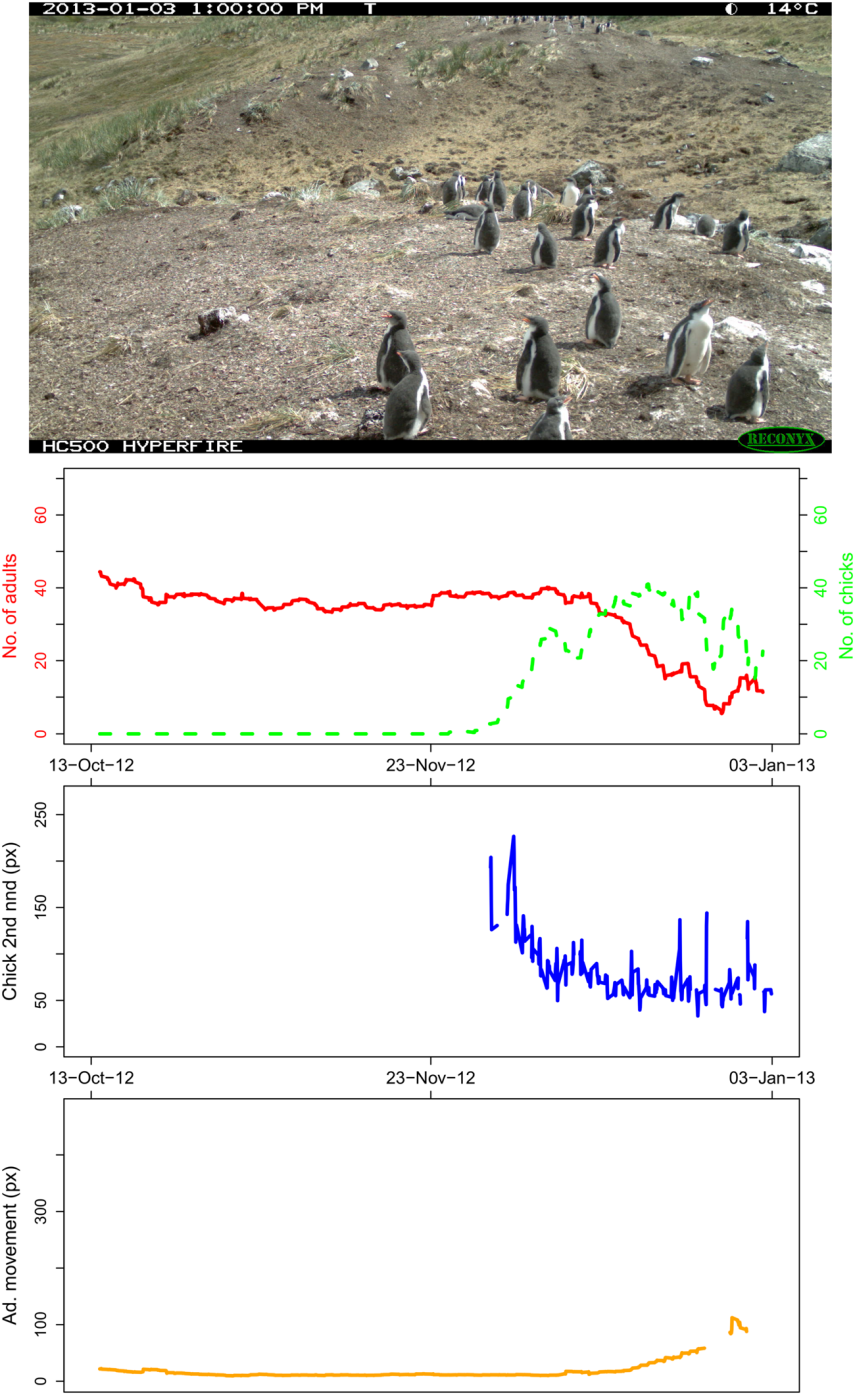
The *Narwhal Plot* for MAIVb2012a_000661 (the last image in the MAIVb2012a data set, therefore showing the complete trends for the data series). The plot comprises, from top down: the original time-lapse image (found in Dataset 1^12^), graph 1: number of adults and chicks (moving average, n = 20), graph 2: average chick ‘second nearest neighbour distances’ (moving average, n = 2), and graph 3: mean adult ‘nearest neighbour distance’ between the *i*th and (*i-1*)th image (moving average, n = 20).

Here we present a second dataset made available through the Dryad Digital Repository (Dataset 2^15^). This contains two main file types. Firstly, files containing data directly derived from *Penguin Watch* citizen science classifications: *Kraken Files* and *Narwhal Files* (Online-only Table 2; produced using the *Kraken Script* and *Narwhal Script*, respectively), *Narwhal Plots* (Fig. 4 and Table 1; generated using the *Narwhal Plotting Script*), and *Penguin Watch Narwhal Animations* (Table 1, produced using GIMP (v2.8)^21^ and VirtualDub (v1.10.4)^22^. Secondly, files produced using computer vision – i.e. the *Pengbot* CNN (albeit initially trained on citizen science dot annotations): *Pengbot Out Files*, *Pengbot Density Maps*, and *Pengbot Count Files* (Table 2). All of these files directly relate to those provided in Dataset 1^12^; i.e. an example within every file type is provided for each of the 63,070 raw time-lapse images captured by 14 cameras, meaning the processing pipeline can be traced for every photograph.

**Table 1:** Available files, and naming conventions, for the processed *Narwhal* data files, plots and animations.

Camera name	*Narwhal File* (.csv)	*Narwhal Plots*	*Penguin Watch Narwhal Animation*
DAMOa	DAMOa_narwhal	DAMOa2014a_nplots	DAMOa2014a_PW_Animation
GEORa	GEORa_narwhal	GEORa2013_nplots	GEORa2013_PW_Animation
HALFb	HALFb_narwhal	HALFb2013a_nplots	HALFb2013a_PW_ Animation
HALFc	HALFc_narwhal	HALFc2013a_nplots	HALFc2013a_PW_ Animation
LOCKb	LOCKb_narwhal	LOCKb2013_nplots	LOCKb2013_PW_ Animation
MAIVb	MAIVb_narwhal	MAIVb2012a_nplots	MAIVb2012a_PW_ Animation
		MAIVb2013a_nplots*	MAIVb2013a_PW_Animation*
		MAIVb2013c_nplots	MAIVb2013c_PW_ Animation
MAIVc	MAIVc_narwhal	MAIVc2013_nplots	MAIVc2013_PW_ Animation
NEKOa	NEKOa_narwhal	NEKOa2012a_nplots	NEKOa2012a_PW_Animation
		NEKOa2013_nplots	NEKOa2013_PW_Animation
		NEKOa2014a_nplots	NEKOa2014a_PW_Animation
NEKOb	NEKOb_narwhal	NEKOb2013_nplots*	NEKOb2013_PW_Animation*
NEKOc	NEKOc_narwhal	NEKOc2013_nplots	NEKOc2013_PW_Animation
		NEKOc2014b_nplots	NEKOc2014b_PW_Animation
PETEc	PETEc_narwhal	PETEc2013_nplots	PETEc2013_PW_ Animation
		PETEc2014_nplots	PETEc2014_PW_ Animation
PETEe	PETEe_narwhal	PETEe2013_nplots	PETEe2013_PW_ Animation
PETEf	PETEf_narwhal	PETEf2014a_nplots	PETEf2014a_PW_ Animation
SPIGa	SPIGa_narwhal	SPIGa2012a_nplots	SPIGa2012a_PW_ Animation
		SPIGa2013b_nplots	SPIGa2013b_PW_ Animation
		SPIGa2014_nplots	SPIGa2014_PW_ Animation

*Missing one plot owing to corruption of raw time-lapse image.

**Table 2:** Available files, and naming conventions, for the *Pengbot* output data.

Camera name	*Pengbot Out Folder* (.matlab)	*Pengbot Density Maps* (.jpg)	*Pengbot Count File* (.csv)
DAMOa	DAMOa_pengbot_out	DAMOa_density_maps	DAMOa_pengbot_count
GEORa	GEORa_pengbot_out	GEORa_density_maps	GEORa_pengbot_count
HALFb	HALFb_pengbot_out	HALFb_density_maps	HALFb_pengbot_count
HALFc	HALFc_pengbot_out	HALFc_density_maps	HALFc_pengbot_count
LOCKb	LOCKb_pengbot_out	LOCKb_density_maps	LOCKb_pengbot_count
MAIVb	MAIVb_pengbot_out	MAIVb_density_maps	MAIVb_pengbot_count
MAIVc	MAIVc_pengbot_out	MAIVc_density_maps	MAIVc_pengbot_count
NEKOa	NEKOa_pengbot_out	NEKOa_density_maps	NEKOa_pengbot_count
NEKOb	NEKOb_pengbot_out	NEKOb_density_maps	NEKOb_pengbot_count
NEKOc	NEKOc_pengbot_out	NEKOc_density_maps	NEKOc_pengbot_count
PETEc	PETEc_pengbot_out	PETEc_density_maps	PETEc_pengbot_count
PETEe	PETEe_pengbot_out	PETEe_density_maps	PETEe_pengbot_count
PETEf	PETEf_pengbot_out	PETEf_density_maps	PETEf_pengbot_count
SPIGa	SPIGa_pengbot_out	SPIGa_density_maps	SPIGa_pengbot_count

Also included in the repository (Dataset 2^15^) are a *Penguin Watch Manifest* file, containing metadata for all of the images, and a *Method Comparison File*, which contains analysis of gold standard, citizen science and *Pengbot* counts for 1183 *Penguin Watch* images (see Technical Validation).

### Explanation of terms

#### Citizen Science Workflow Files

##### *Penguin Watch Manifest name:*

 Unique image reference for identification, in the format: SITExYEARx_imagenumber; e.g. DAMOa2014a_000001.

*datetime:* Time and date information for the image, in the format YYYY:MM:DD HH:MM:SS.

*zooniverse_id:* Unique identification code assigned to each image within Zooniverse (the online platform that hosts *Penguin Watch* – see www.zooniverse.org).

*path:* Folder pathway, which includes the image name (e.g. DAMOa/DAMOa2014a_000025).

*classification_count:* Number of volunteers who classified the image before it was retired.

*state:* State of completion – either complete (the image has been shown to the required number of volunteers) or incomplete (the image requires classification by further volunteers).

*temperature_f:* Temperature (in degrees Fahrenheit, as recorded by the camera) at the time the photograph was taken.

*lunar_phase:* Moon phase when the image was captured (one of eight options: “full” (full), “new” (new), “newcres” (new crescent), “firstq” (first quarter), “waxinggib” (waxing gibbous), “waninggib” (waning gibbous), “lastq” (last quarter) or “oldcres” (old crescent)).

*URL:* Link to an online thumbnail image of the time-lapse photograph (lower resolution than the raw time-lapse imagery included in the repository, but useful for reference).

##### *Kraken Files*

*Kraken Files* comprise filtered ‘consensus clicks’ and metadata (Online-only Table 2). The filtering threshold levels are ‘*num_markings* > *3’* for adults and ‘*num_markings* > *1’* for chicks and eggs (see Methods). Each row contains the following information. Please note that where ‘NA’ is given for probability values, ‘num_markings’, ‘x_centre’ and ‘y_centre’, no penguins have been identified in the image.

*name:* Unique image reference for identification, in the format: SITExYEARx_imagenumber; e.g. DAMOa2014a_000001.

*probability_of_adult:* Estimated probability that the corresponding individual is an adult – based on the number of volunteers classifying it as such, as a proportion of the total number of clicks on that individual.

*probability_of_chick:* Estimated probability that the corresponding individual is a chick – based on the number of volunteers classifying it as such, as a proportion of the total number of clicks on that individual.

*probability_of_egg:* Estimated probability that the corresponding marking indicates an egg – based on the number of volunteers classifying it as such, as a proportion of the total number of clicks on that area.

*num_markings:* The number of volunteer clicks that were aggregated to produce the ‘consensus click’ coordinate values (i.e. the number of individual clicks on a specific area of the image). Owing to the filtering process, *num_markings* is ‘*>3*’ for adults and ‘*>1*’ for chicks and eggs in the files provided alongside this Data Descriptor. These threshold levels can be changed in the *Kraken Script*, to create *Kraken Files* with different degrees of filtering.

*x_centre:* x coordinate value (in pixels) for the ‘consensus click’ (i.e. the coordinate calculated by the clustering algorithm)^10,14^. The origin (point 0, 0) is located in the top left-hand corner of the image, meaning it may be necessary to reverse the y-axis of a plot in order to overlay the ‘consensus clicks’ correctly. One coordinate value denotes one individual penguin/‘other’.

*y_centre:* y coordinate value (in pixels) for the ‘consensus click’ (i.e. the coordinate calculated by the clustering algorithm)^10,14^. The origin (point 0, 0) is located in the top left-hand corner of the image, meaning it may be necessary to reverse the y-axis of a plot in order to overlay the ‘consensus clicks’ correctly. One coordinate value denotes one individual penguin/‘other’.

For datetime, temperature_f, lunar_phase and URL please see ‘Penguin Watch Manifest’.

##### *Narwhal Files*

*Narwhal Files* contain penguin count data, ‘nearest neighbour distance’ metrics, and metadata. Each row contains the following information (Online-only Table 2):

*imageid:* Unique image reference for identification, in the format: SITExYEARx_imagenumber; e.g. DAMOa2014a_000001.

*nadults:* The total number of adults counted in the corresponding image, based on filtered (here, *num_markings > 3*) ‘consensus click’ data (see *Kraken Files*).

*nchicks:* The total number of chicks counted in the corresponding image, based on filtered (here, *num_markings > 1*) ‘consensus click’ data (see *Kraken Files*).

*neggs:* The total number of eggs counted in the corresponding image, based on filtered (here, *num_markings > 1*) ‘consensus click’ data (see *Kraken Files*).

*adultndout:* The mean adult ‘nearest neighbour distance’. Calculated by taking the distance between each adult and its nearest (adult) neighbour, and finding the mean average.

*adultsdndout:* Standard deviation of the adult ‘nearest neighbour distances’.

*chickndout:* The mean chick ‘nearest neighbour distance’. Calculated by taking the distance between each chick and its nearest (chick) neighbour, and finding the mean average.

*chicksdndout:* Standard deviation of the chick ‘nearest neighbour distances’.

*chick2ndout:* The mean distance between each chick and its second nearest (chick) neighbour within each image. The second nearest neighbour is calculated because the *Pygoscelis* penguins (the primary focus of the *Penguin Watch* camera network) usually lay two eggs. Since a chick’s nearest neighbour is therefore likely to be its sibling in the nest, the distance to the second nearest neighbour is calculated, to provide more information about the spatial distribution of chicks within the colony.

*chicksd2ndout:* Standard deviation of the chick ‘second nearest neighbour distances’.

*meanchangeadult:* Movement of each adult [*j*] between image [*i-1*] and image [*i*]. This is calculated by appending the xy coordinate of adult [*j*] in image [*i*] to a dataframe of the adult xy coordinates in image [*i-1*], and calculating the ‘nearest neighbour distance’ for adult [j]. The nearest adult to [*j*] is likely itself in the previous image, therefore this distance represents movement between the two images. It is possible that the nearest neighbour is a different individual, so a mean average is calculated to provide an indication of movement within the field of view. This metric is most appropriate between incubation and the end of the guard phase, when adults are often at the nest (see Methods).

*meanchangechick:* Movement of each chick [*k*] between image [*i-1*] and image [*i*]. This is calculated by appending the xy coordinate of chick [*k*] in image [*i*] to a dataframe of the chick xy coordinates in image [*i-1*], and calculating the ‘nearest neighbour distance’ for chick [*k*]. The nearest chick to [*k*] is likely itself in the previous image, therefore this distance represents movement between the two images. It is possible that the nearest neighbour is a different individual, so a mean average is calculated to provide an indication of movement within the field of view. This metric is most appropriate between incubation and the end of the guard phase, when chicks are at the nest (see Methods).

*tempf:* Temperature (in degrees Fahrenheit, as recorded by the camera) at the time the photograph was taken.

*tempc:* Temperature (in degrees Celsius) at the time the photograph was taken.

For datetime, lunar_phase and URL, please see ‘Penguin Watch Manifest’

##### *Narwhal Plots and Penguin Watch Narwhal Animations*

*Narwhal Plots* (see Fig. 4), generated using the *Narwhal Plotting Script*, provide a visualisation of summary statistics: graph 1: abundance of adults and chicks; graph 2: chick ‘second nearest neighbour distances’; and graph 3: mean adult ‘nearest neighbour distance’ between the *i*th and (*i-1*)th image. A plot is produced for each time-lapse image (Table 1), showing the moving average trends up to, and including, that image. Therefore, to see the complete trend, a plot should be created for the final image in the data series. The complete sets of graphs have been used to create *Penguin Watch Narwhal Animations*, which show the trends developing through time, alongside the time-lapse images. The folders of *Narwhal Plots* and *Penguin Watch Narwhal Animations* correspond to those described in Table 4 of Jones *et al*. (2018) (with the exception of PETEd2013, which is not included as it is a duplicate image set of PETEc2014).

#### Computer Vision (Pengbot) Files

##### *Pengbot Out Files*

*Pengbot Out Files* are MATLAB files containing a matrix of ‘penguin densities’, calculated using the *Pengbot* model. A density value is provided for each pixel in the corresponding raw time-lapse photograph. A file is provided (in Dataset 2^15^) for each of the 63,070 images presented in Dataset 1^12^; they are separated into folders according to camera (e.g. DAMOa_pengbot_out) (Table 2).

##### *Pengbot Density Maps*

A *Pengbot Density Map* (Dataset 2^15^), generated using the *Pengbot Counting Script* (see Code Availability), is provided for each of the 63,070 raw time-lapse photographs presented in Dataset 1^12^. The maps are a visual representation of the pixel densities presented in the *Pengbot Out Files*. Maps are stored in the database according to camera (e.g. DAMOa_density_map) (Table 2; Fig. 3, right).

##### *Pengbot Count Files*

These are generated using the *Pengbot Counting Script* (see Code Availability). They provide a penguin count (adults and chicks combined) for each image by summing the ‘penguin density’ of each pixel in the *Pengbot Out Files* (Table 2).

*imageid:* Unique image reference for identification, in the format: SITExYEARx_imagenumber; e.g. DAMOa2014a_000001.

*raw_count:* The number of penguins in the image, as calculated by the *Pengbot* model^11^. Since the counts are generated by summing pixel densities, they are unlikely to be an integer value.

*count*: The number of penguins in the image as calculated using *Pengbot*, rounded to the nearest integer.

#### Method comparison file

This file contains penguin counts for 1183 images, from four different cameras – DAMOa (Damoy Point; n = 300), HALFc (Half Moon Island; n = 283), LOCKb (Port Lockroy; n = 300) and PETEc (Petermann Island; n = 300) – as calculated by an expert (‘Gold Standard’), through *Penguin Watch* (‘Citizen Science’), and by *Pengbot* (‘Computer Vision’).

*imageid:* Unique image reference for identification, in the format: SITExYEARx_imagenumber; e.g. DAMOa2014a_000001.

*datetime:* Time and date information for the image, in the format YYYY:MM:DD HH:MM:SS.

*GS_adults:* The number of penguin adults in the corresponding image, as identified and calculated by an expert.

*GS_combined:* The number of penguin adults and chicks in the corresponding image, as identified and calculated by an expert.

*CS_adults:* The number of penguin adults in the corresponding image, generated using clustered citizen science data from the *Penguin Watch* project (threshold = *num_markings* >* 3*).

*CS_combined:* The number of penguin adults and chicks, as generated using clustered citizen science data from the *Penguin Watch* project (threshold = *num_markings* >* 3* for adults and *num_markings* >* 1* for chicks).

*CV_rounded:* The rounded number of penguin individuals (adults and chicks combined) in the corresponding image, calculated using the *Pengbot* model^11^.

The file also includes columns for six comparisons:

GS_combined vs. CV_rounded; GS_adults vs. CV_rounded; CS_combined vs. CV_rounded; CS_adults vs. CV_rounded; GS_combined vs. CS_combined; GS_adults vs. CS_adults.

Two columns are provided for each comparison. The first column shows the difference (in raw number of penguins) between the counts generated via the two methods. For example, if the gold standard combined count was 15 and the computer vision count was 12, then the ‘GS_combined vs. CV_rounded’ column would contain the value 3. The second column shows the same values, with any negative signs removed (whether the counts are under- or over-estimates is irrelevant when calculating average differences).

The average difference in counts, the standard deviation of this difference, the proportion of counts that are equal or differ by only one penguin, and the number of over- and under-estimates are also provided in the spreadsheet (and summarised in Table 3). Overestimates and underestimates relate to the second variable, i.e. for ‘GS_combined vs. CV_rounded’, an ‘underestimate’ would mean an underestimate by computer vision, as compared to the gold standard.

**Table 3:** Comparison between gold standard (GS; expert classifications), citizen science (CS*; Penguin Watch*) and computer vision (CV; *Pengbot*) counts (n = 1183).

	GS vs. CV (combined)	GS vs. CV (adults)	CS vs. CV (combined)	CS vs. CV (adults)	GS vs. CS (combined)	GS vs. CS (adults)
**DAMOa**	n = 300					
Average difference	1.95	2.01	2.21	2.25	1.36	1.18
σ	1.84	2.15	2.16	2.38	1.66	1.56
Proportion 0 or 1	0.49	0.49	0.47	0.47	0.70	0.75
Overestimate	121	143	116	145	102	101
Underestimate	115	90	136	102	113	100
**HALFc**	n = 283					
Average difference	1.50	1.21	1.46	1.45	1.20	0.88
σ	1.36	1.08	1.70	1.56	1.53	1.34
Proportion 0 or 1	0.61	0.69	0.65	0.66	0.72	0.85
Overestimate	60	107	70	109	58	64
Underestimate	161	101	134	99	116	91
**LOCKb**	n = 300					
Average difference	1.83	3.71	2.13	3.50	1.23	1.17
σ	1.70	4.27	2.50	4.10	2.03	1.98
Proportion 0 or 1	0.55	0.45	0.52	0.46	0.72	0.77
Overestimate	68	157	80	159	61	82
Underestimate	168	96	151	81	92	80
**PETEc**	n = 300					
Average difference	12.13	7.17	11.31	7.09	3.70	2.36
σ	5.07	3.39	7.19	4.17	4.12	2.69
Proportion 0 or 1	0	0.06	0.02	0.08	0.36	0.46
Overestimate	0	81	1	84	85	79
Underestimate	300	212	297	207	170	162

Average differences (in raw count) are provided, alongside the standard deviation of these differences, the proportion of counts that were equal or differed by only one penguin, and the number of over- and underestimates. For GS and CS, combined counts (i.e. adults and chicks) and adult-only counts are included. The filtering threshold levels used for the CS counts are *num_markings* > *3* for adults and *num_markings* > *1* for chicks. CV cannot distinguish between adults and chicks, so a single value is provided. Overestimates and underestimates relate to the second variable, i.e. for DAMOa ‘GS vs. CV (combined)’, computer vision overestimated the penguin count in 121 cases, and underestimated it in 115 cases, as compared to the gold standard. GS vs CS counts are included for completeness – please see Jones *et al*. (2018) for a full discussion.

## Technical Validation

In Jones *et al*. (2018) we provide a technical validation of *Penguin Watch* citizen science data, comparing clustered counts (at four different threshold levels) to counts derived from expert annotations (the ‘gold standard’). Here we extend this analysis by comparing counts generated by the *Pengbot* computer vision algorithm to both gold standard and citizen science count data (see Table 3).

As in Jones *et al*. (2018), images from cameras at four sites (Damoy Point (DAMOa2014a), Half Moon Island (HALFc2013a), Port Lockroy (LOCKb2013) and Petermann Island (PETEc2013)) were employed in the analysis, to ensure that images showing different camera angles and colony proximity, and capturing all three *Pygoscelis* species, were included. Gold standard classifications (annotations by author FMJ) and citizen science counts were taken from Jones *et al*. (2018), where a sample of 300 images was randomly selected for each site from the photographs that were (1) marked as containing animals and (2) were marked as complete in *Penguin Watch*. (Note that an exception to this is HALFc, where the whole sample of 283 images was used, to give a total of 1183).

Here, citizen science counts generated using a clustering threshold level of >3 for adults and >1 for chicks (i.e. four or more volunteer clicks, or two or more volunteer clicks, were required to form a ‘consensus click’, respectively) are used, since these filtering levels were previously found to produce counts most similar to the gold standard, in most cases (see Jones *et al*., 2018). These are also the threshold levels used to create the *Kraken* and *Narwhal Files* discussed in this Data Descriptor. Adult counts and combined counts (i.e. adults and chicks) are provided for the gold standard and citizen science methods, to examine the impact of this on the agreement with computer vision, which cannot yet distinguish between these groups.

**Fig. 5:**
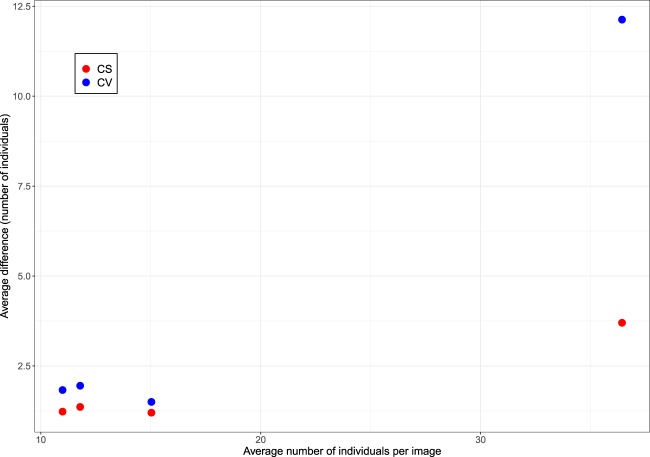
The average difference between GS and CV counts (blue) and GS and CS counts (red), shown against the average number of individuals per image. A = DAMOa, B = HALFc, C = LOCKb, D = PETEc.

The average difference between the gold standard (combined) and computer vision derived counts was less than two for DAMOa, HALFc and LOCKb (1.95 (σ = 1.84), 1.50 (σ = 1.36) and 1.83 (σ = 1.70), respectively) – similar to the differences between gold standard (combined) and citizen science (combined) counts (see Fig. 5). The degree of error was greater for PETEc, where the count differed by 12.13 penguins on average. The greatest discrepancies between the gold standard and citizen science counts were also associated with this site (likely owing to the higher average number of adults and chicks at this site – see Jones *et al*., 2018).

With the exception of DAMOa, the number of underestimates outweighed the number of overestimates for each site, showing that the discrepancies were mainly owing to ‘missed’ individuals. As discussed in Jones *et al*. (2018), chicks are often missed by citizen science volunteers – perhaps owing to obscuration by an adult, misidentification, or – since volunteers can move onto another image after marking 30 individuals – simply going unannotated. While the latter is irrelevant for computer vision, it is possible that chicks go undetected by the algorithm owing to their small size, and position underneath (or directly in front of) a parent.

**Fig. 6:**
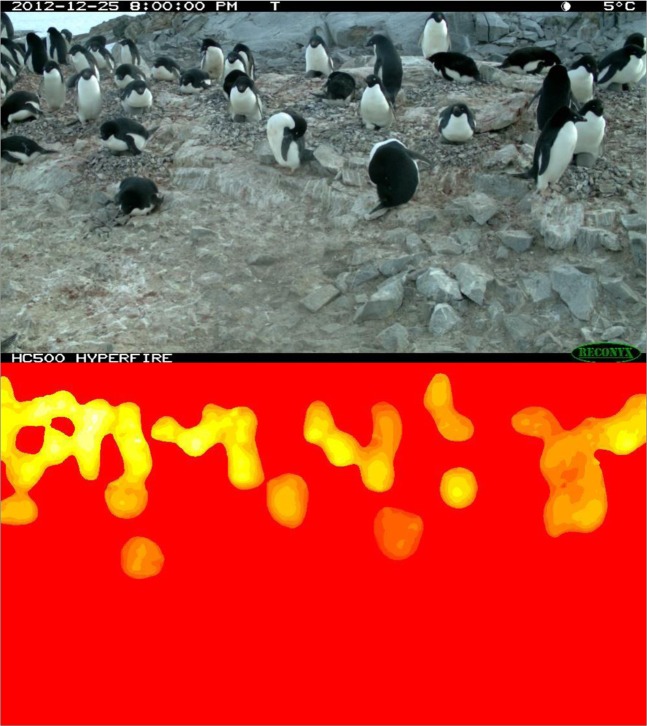
Image data from an Adélie Penguin (*Pygoscelis adeliae*) colony at Petermann Island, Antarctic Peninsula, on 25/12/2012 at 20:00:00. The raw time-lapse image (top) and density map (bottom) for PETEc2013b_000023 show how crowding and occlusion can lead to underestimation by *Pengbot*. While the computer vision algorithm is correctly identifying ‘penguin pixels’, the degree of occlusion – particularly in the top left-hand corner of the image – makes counting a challenge.

Since the proportion of underestimates generally increases with an increasing average number of chicks per image, it is possible that undetected chicks are a notable contributor to the error rate. However, while the proportion of overestimates is increased when chick counts are excluded (as expected, since the computer vision counts remain constant), the overall agreement is not improved in all cases (i.e. for DAMOa and LOCKb). Therefore, there are other factors contributing to the high proportion of underestimated counts. As stated, the largest discrepancies between the computer vision derived counts and gold standard counts are associated with PETEc, where 100% of counts were underestimates when compared to the gold standard (combined). This is also the site which has the highest number of individuals per image on average (36.43 compared to 11.79, 15.03 and 10.99 at DAMOa, HALFc and LOCKb, respectively). A greater number of individuals leads to a higher probability of crowding, with some individuals occluded by others (see Fig. 6). While the human eye can detect a partially-obscured penguin (in an extreme case, being able to mark an individual when only its beak is visible), the computer vision algorithm – which counts penguins by summing pixel densities – cannot achieve this. Furthermore, a problem which has the potential to affect all images is an “edge-effect”. Again, if only half a penguin is visible on the perimeter of an image, a human annotator will record this as ‘one’ penguin. However, the computer vision algorithm can only record it as half an individual, lowering the overall estimation.

**Fig. 7:**
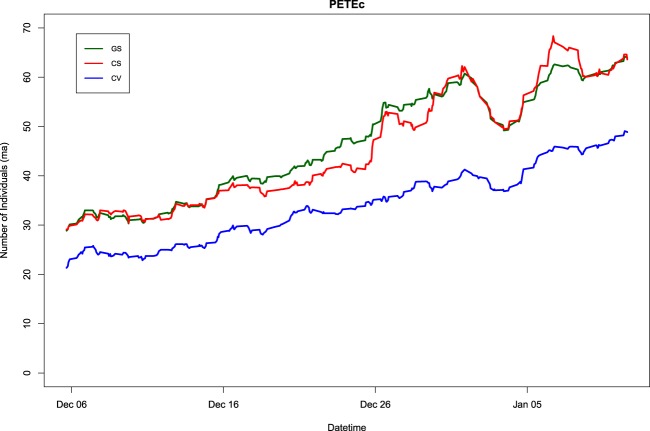
The penguin population trend at Petermann Island, Antarctic Peninsula (camera PETEc) between 3/12/2012 and 11/01/2013, as determined using expert-annotations (gold standard (GS); green), *Penguin Watch* (citizen science (CS); red), and the *Pengbot* algorithm (computer vision (CV); blue). Trends are shown as a moving average (n = 15).

These three computer vision issues – missed chicks, occlusion of individuals, and the edge-effect – likely act in combination to produce underestimates of counts, particularly when images contain high numbers of individuals, such as at PETEc. However, as shown by Table 3 and Fig. 5, computer vision offers a valid alternative to expert annotation or citizen science when examining images with fewer individuals. Moreover, when investigating colony phenology, overall population trends are of greater interest than raw population counts. As shown in Fig. 7, the main population trends observed at PETEc are preserved irrespective of the annotation method used, justifying the use of both citizen science and computer vision for this task.

One way of using computer vision and citizen science in combination would be to introduce a pipeline where images are first processed using computer vision, and then only uploaded to *Penguin Watch* if penguins have been detected. However, while it might be assumed that blank images would cause boredom in volunteers, a study by Bowyer *et al*. (2016) shows the opposite to be true. When a higher percentage of blank images were introduced into the *Snapshot Serengeti*^23^ citizen science project, mean average session length (the number of images seen by a volunteer) also increased^24^. This suggests that volunteers are motivated by the excitement of ‘discovery’ (see the “intermittent-reinforcement” theory^25^), and supports the inclusion of blank images in the *Penguin Watch* project^24^.

Remote camera technologies have the potential to monitor animal and plant populations in locations which may otherwise be impossible to effectively survey. Here we show that citizen science and computer vision offer alternative, albeit complementary, approaches to image analysis, meaning that the vast quantity of data produced via camera networks should not be a barrier to their use. We demonstrate how meaningful biological metrics can be extracted from imagery, and hope that the *Penguin Watch* data presented here may form a case study for those wishing to carry out similar analyses, and may be useful to both ecologists and computer vision developers.

## Data Availability

Code used to produce files within Dataset 1^12^: • Raw *Penguin Watch* time-lapse photographs are renamed and resized using an *R* (currently v3.6.0) script. The code is publically available via GitHub at https://github.com/zooniverse/Data-digging/blob/master/example_scripts/Penguin_Watch/Penguin_Watch_ImageProcessingScript.R. A static version (written using v3.4.1) is archived on Figshare^26^. • Raw *Penguin Watch* volunteer classifications (xy coordinate clicks) were clustered into ‘consensus clicks’ using agglomerative hierarchical clustering (Fig. 1, left, ‘Step 2’)^10,14^. The aggregation algorithm is written in Python (v 2.7) and can be found on GitHub at https://github.com/zooniverse/aggregation/blob/master/penguins/aggregate.py. A static version of this script is also archived on Figshare^14^. Code used to produce files within Dataset 2^15^: • The *Kraken Script* (Fig. 1, left, ‘Step 3’; output = *Kraken Files*) is written in *R* (v3.6.0); it can be accessed through GitHub at https://github.com/zooniverse/Data-digging/blob/master/example_scripts/Penguin_Watch/Kraken_Script.R, and a static version is archived on Zenodo^27^. • The *Narwhal Script* (Fig. 1, left, ‘Step 4’; output = *Narwhal Files*) is written in *R* (v3.6.0); it can be accessed through GitHub at https://github.com/zooniverse/Data-digging/blob/master/example_scripts/Penguin_Watch/Narwhal_Script.R and a static version is archived on Zenodo^28^. • The *Narwhal Plotting Script* (output = *Narwhal Plots* – graphs displaying *Narwhal* summary statistics) is written in *R* (v3.6.0); it can be accessed through GitHub at https://github.com/zooniverse/Data-digging/blob/master/example_scripts/Penguin_Watch/Narwhal_Plotting_Script.R and a static version is archived on Zenodo^29^. • The *Pengbot* model^11^, associated code and instructions, and the dataset used to train the neural network can be found at the following address: https://www.robots.ox.ac.uk/~vgg/data/penguins/. • The *Pengbot Counting Script* (Fig. 1, right, ‘Step 2’ and ‘Step 3’) is written in *R* (v3.6.0); it can be accessed through GitHub at https://github.com/zooniverse/Data-digging/blob/master/example_scripts/Penguin_Watch/Pengbot_Counting_Script.R and a static version is archived on Zenodo^30^.

## Online-only Tables

**Online-only Table 1:** Explanation of variables within the *Narwhal Files*, and the code used to produce them.

Variable in *Narwhal File*	*R* code (in *Narwhal Script*) to calculate (for the *i*th value)	Explanation of variable
nadults	length (currentadult [1])	Number of adults. The length (number of rows) of the ‘adult’ ‘consensus click’ data (clustered xy coordinates) associated with the current image.
nchicks	length (currentchick [1])	Number of chicks. The length (number of rows) of the ‘chick’ ‘consensus click’ data (clustered xy coordinates) associated with the current image.
neggs	length (currenteggs [1])	Number of eggs. The length (number of rows) of the ‘egg’ ‘consensus click’ data (clustered xy coordinates) associated with the current image.
adultndout	mean(nndist(currentadultxy)	Mean ‘nearest neighbour distance’ between each adult in image [*i*] and its nearest neighbour.
adultsdndout	sd (nndist (currentadultxy))	Standard deviation of the adult ‘nearest neighbour distances’.
chickndout	mean (nndist (currentchickxy))	Mean ‘nearest neighbour distance’ between each chick in image [*i*] and its nearest neighbour.
chicksdndout	sd (nndist (currentchickxy))	Standard deviation of the chick ‘nearest neighbour distances’.
chick2ndout	mean (nndist (currentchickxy, k = 2))	The mean distance between each chick and its second nearest neighbour within each image. The second nearest neighbour is calculated to account for the fact that most nests have two chicks, so the first nearest neighbour is likely to be a sibling (and will therefore be less informative regarding movement in the colony).
chicksd2ndout	sd (nndist (currentchickxy, k = 2))	Standard deviation of the chick ‘second nearest neighbour distances’.
meanchangeadult	Create a data frame of xy coordinates for each adult [*j*] for each image [*i*], and the previous image [*i-1*]: currentadultxy < -data.frame(currentadult$x_centre, currentadult$y_centre) prevadultxy < -data.frame(prevadult$x_centre, prevadult$y_centre) Add adult [*j*] in image [*i*] to the dataframe for image [*i-1*]: prevadultx < -c(currentadultx, prevadult$x_centre) prevadulty < -c(currentadulty, prevadult$y_centre) prevadultxy < -data.frame(prevadultx, prevadulty) Calculate nnd for just the adult of interest [*j*]: temp < -nndist(prevadultxy)) prevadultndout[*j*] < -temp[1] Calculate the mean of these nnds: meanchangeadult[*i*] < -mean(prevadultndout)	The mean distance between each adult [*j*] in image [*i*] and its nearest neighbour in image [*i-1*]. This means the coordinates of adult [*j*] must be appended to all the adult coordinates in the previous image, to make a new dataframe ([*i-1*] with one additional data point – adult [*j*] in image [*i*]). The same adult will thus be represented by two data points in this new dataframe. ‘Nearest neighbour distance’ is then calculated as before, but only for the adult of interest [*j*]. It is likely that adult [*j*]’s nearest neighbour will be itself, in its previous position, meaning this value is equivalent to the distance adult [*j*] has moved between images. The mean is calculated to give the average distance moved by all adults in the image.
meanchangechick	Create a data frame of xy coordinates for each chick [*k*] for each image [*i*], and the previous image [*i-1*]: currentchickxy < -data.frame(currentchick$x_centre, currentchick$y_centre) prevchickxy < -data.frame(prevchick$x_centre, prevchick$y_centre) Add chick [*k*] in image [*i*] to the dataframe for image [*i-1*]: prevchickx < -c(currentchickx, prevchick$x_centre) prevchicky < -c(currentchicky, prevchick$y_centre) prevchickxy < -data.frame(prevchickx, prevchicky) Calculate nnd for just the chick of interest [*k*]: temp2 < -nndist(prevchickxy)) prevchickndout[*k*] < -temp2[1] Calculate the mean of these nnds: meanchangechick[*i*] < -mean(prevchickndout)	The mean distance between each chick [*k*] in image [*i*] and its nearest neighbour in image [*i-1*]. This means the coordinates of chick [*k*] must be appended to all the chick coordinates in the previous image, to create a new dataframe ([*i-1*] with one additional data point – chick [*k*] in image [*i*]). The same chick will thus be represented by two data points in this new dataframe. ‘Nearest neighbour distance’ is then calculated as before, but only for the chick of interest [*k*]. It is likely that the nearest neighbour to chick [*k*] will be itself in its previous position, meaning this value is equivalent to the distance chick [*k*] has moved between images. The mean is calculated to give the average distance moved by all chicks in the image.

**Online-only Table 2:** Information relating to the *Kraken* and *Narwhal* Files stored in Dataset 2.

Site (camera location)	Camera name	Image set (name in the Dataset 1^12^)	Time-range	*Kraken File* (csv)	*Narwhal File* (csv)
Damoy Point, Weincke Island, Antarctic Peninsula; 64.82° S, 63.49° W	DAMOa	DAMOa2014a	26/12/2013 1300 to 22/01/2014 1400 (0700–2100; hourly)	DAMOa_kraken	DAMOa_narwhal
George’s Point, Rongé Island on the Errera Channel, Antarctic Peninsula; 64.67° S, 62.67° W	GEORa	GEORa2013	22/12/2012 1000 to 24/12/2013 0800 (0800–1900; hourly)	GEORa_kraken	GEORa_narwhal
Half Moon Island, South Shetland Islands; 62.60° S, 59.90° W	HALFb	HALFb2013a	15/12/2012 1700 to 08/01/2013 1000 (0700–1900; hourly)	HALFb_kraken	HALFb_narwhal
	HALFc	HALFc2013a	21/12/2012 1700 to 09/01/2013 1400 (0700–2100; hourly)	HALFc_kraken	HALFc_narwhal
Port Lockroy, Wiencke Island, Antarctic Peninsula; 64.82° S, 63.49° W	LOCKb	LOCKb2013	13/12/2012 1100 to 31/05/2013 1600 (0700–2000; hourly)	LOCKb_kraken	LOCKb_narwhal
Maiviken, South Georgia; 54.24° S, 36.50° W	MAIVb	MAIVb2012a	13/10/2012 0900 to 03/01/2013 1300 (0900–1100, 1300–1700; hourly)	MAIVb_kraken	MAIVb_narwhal
		MAIVb2013a	03/01/2013 1400 to 28/10/2013 1700 (0800–2000; hourly)		
		MAIVb2013c	29/10/2013 0800 to 08/11/2013 1400 (0800–2000; hourly)		
	MAIVc	MAIVc2013	15/10/2012 0900 to 08/11/2013 1300 (0900–1100, 1300–1700; hourly)	MAIVc_kraken	MAIVc_narwhal
Neko Harbour, Andvord Bay, Antarctic Peninsula; 64.86° S, 62.52° W	NEKOa	NEKOa2012a	03/03/2012 1000 to 25/11/2012 1500 (1000–1500; hourly)	NEKOa_kraken	NEKOa_narwhal
		NEKOa2013	26/11/2012 1000 to 25/12/2013 1500 (0800–1600; hourly)		
		NEKOa2014a	25/12/2013 1600 to 13/01/2014 0700 (0700–1900; hourly)		
	NEKOb	NEKOb2013	02/03/2012 1600 to 26/11/2012 0800 (0000–1200; hourly)	NEKOb_kraken	NEKOb_narwhal
	NEKOc	NEKOc2013	14/12/2012 1600 to 19/11/2013 1000 (0700–1900; hourly)	NEKOc_kraken	NEKOc_narwhal
		NEKOc2014b	(25/12/2013 1600–13/01/2014 0700) (0600–2100; hourly)		
Petermann Island, Antarctic Peninsula; 65.17° S, 64.14° W	PETEc	PETEc2013	03/12/2012 1600 to 11/01/2013 1400 (0800–1100, 1300–2100; hourly)	PETEc_kraken	PETEc_narwhal
		PETEc2014	11/01/2013 1430 to 04/01/2014 1000 (0700–2130; half hourly)		
	PETEe	PETEe2013	12/12/2012 1600 to 04/01/2014 0900 (0900–1800; hourly)	PETEe_kraken	PETEe_narwhal
	PETEf	PETEf2014a	24/12/2012 1300 to 04/01/2014 1000 (0100–1100, 1300–2300; hourly)	PETEf_kraken	PETEf_narwhal
Spigot Peak, Orne Harbour, Antarctic Peninsula; 64.63° S, 62.57° W	SPIGa	SPIGa2012a	25/11/2012 1700 to 21/12/2012 1600 (0700–2100; hourly)	SPIGa_kraken	SPIGa_narwhal
		SPIGa2013b	09/01/2013 1500 to 23/12/2013 1500 (0700–2100; hourly)		
		SPIGa2014	23/12/2013 1600 to 21/01/2014 2000 (0700–2000; hourly)		
